# The Metabolic Syndrome Is Associated With Lower Cognitive Performance and Reduced White Matter Integrity in Midlife: The CARDIA Study

**DOI:** 10.3389/fnins.2022.942743

**Published:** 2022-07-18

**Authors:** Christina S. Dintica, Tina Hoang, Norrina Allen, Stephen Sidney, Kristine Yaffe

**Affiliations:** ^1^Department of Psychiatry and Behavioral Sciences, University of California, San Francisco, San Francisco, CA, United States; ^2^Northern California Institute for Research and Education, San Francisco, CA, United States; ^3^San Francisco Veterans Affairs Medical Center, San Francisco, CA, United States; ^4^Department of Preventive Medicine, Northwestern University Feinberg School of Medicine, Chicago, IL, United States; ^5^Kaiser Permanente Northern California, Oakland, CA, United States

**Keywords:** metabolic syndrome, cognition, neuroimaging, midlife, diffusion tension imaging (DTI)

## Abstract

**Background:**

Cardiovascular disease risk factors play a critical role in brain aging. The metabolic syndrome (MetS), a constellation of cardiovascular risk factors, has been associated with poorer cognition in old age; however, it is unclear if it is connected to brain health earlier in life.

**Methods:**

We investigated the association of MetS (*n* = 534, 18.5%) vs. no MetS (*n* = 2,346, 81.5%) with cognition in midlife within the prospective study, Coronary Artery Risk Development in Young Adults (CARDIA). At midlife (mean age 50), MetS was defined using National Cholesterol Education Program guidelines. At the 5-year follow-up, a cognitive battery was administered including tests of processing speed (Digit Symbol Substitution Test, DSST), executive function (the Stroop Test), verbal memory (Rey Auditory Verbal Learning Test, RAVLT), verbal fluency (category and letter fluency), and global cognitive function (Montreal Cognitive Assessment, MoCA). A sub-sample (*n* = 453) underwent brain MRI.

**Results:**

Participants with MetS had worse performance on tests of verbal fluency, processing speed, executive function, and verbal memory (*p* < 0.05), but not on global cognition. MetS was also associated with lower frontal, parietal, temporal, and total white matter integrity (*p* < 0.05), as assessed with fractional anisotropy.

**Conclusions:**

MetS is associated with lower cognition and microstructural brain alterations already at midlife, suggesting that MetS should be targeted earlier in life in order to prevent adverse brain and cognitive outcomes.

## Introduction

Early identification and management of modifiable risk factors for cognitive decline and dementia are key in order to delay onset and progression (Petersen et al., 2006). Evidence has shown that cardiovascular risk factors (CVRFs) increase the risk of ischemic brain changes, cognitive impairment, and dementia (Whitmer et al., 2005; Yaffe et al., 2007; Romero et al., 2009; The SPRINT MIND Investigators for the SPRINT Research Group, 2019). However, the influence of CVRFs on brain health earlier in life has not been well studied, particularly at a population level. A prime candidate for early intervention is the metabolic syndrome (MetS) because it encompasses a constellation of CVRFs such as dyslipidemia, hypertension, hyperglycemia, and visceral obesity, which are associated with higher risk of cardiovascular disease, mortality, and other adverse health outcomes (Alberti et al., 2005). Thus, interventions that ameliorate multiple of these CVRFs at once may yield greater disease-specific prevention.

The MetS has been associated with increased risk for vascular dementia (Whitmer et al., 2005) and cognitive impairment in older adults (Yaffe et al., 2009). Moreover, research on CVRFs and cognitive function suggests that risk factors operate in a dose-dependent manner, with likelihood of later-life dementia increasing with greater number of risk factors (Whitmer et al., 2005). While studies on older adults have shown that MetS increases the risk for accelerated cognitive decline in late life (Yaffe et al., 2007, 2009), it is not clear if this association may be present in midlife.

A potential mechanism for the association between MetS and cognitive dysfunction is the presence of microvascular alterations that lead to white matter (WM) disruption. There is growing evidence suggesting an association between MetS and impairment of WM integrity and microstructural damage (Shimoji et al., 2013; Alfaro et al., 2016). However, most of these studies have been case-control studies or observational studies with wide age ranges, hence limiting the interpretation of such findings at population level and during midlife (Alberti et al., 2005; Yaffe et al., 2009; Shimoji et al., 2013). The aim of this study was to investigate the association of MetS with cognitive function and white matter integrity at midlife.

## Methods

### Study Population

The Coronary Artery Risk Development in Young Adults (CARDIA) is a prospective cohort study investigating the development of and risk factors for cardiovascular disease (Friedman et al., 1988). Young adults between 18 and 30 years of age were recruited from population-based samples of 4 US cities (Birmingham, AL; Chicago, IL; Minneapolis, MN; and Oakland, CA). Participants completed the follow-up examinations every 2–5 years for 30 years from 1987 to 2016. At each examination, the participants provided written informed consent, and study protocols were reviewed by institutional review boards at each study site, the CARDIA Coordinating Center each study site. Further details regarding the design and recruitment of CARDIA have been previously reported elsewhere (Friedman et al., 1988).

To assess the effect of MetS on cognitive performance in midlife, we included participants who had MetS assessment at year 25 (2010–2011) *n* = 3,498, our study baseline, and completed a cognitive testing 5 years later (2015–2016), *n* = 2,880 participants. Out of the analytical cohort, 453 participants had MRI data available at 2015–2016.

### Ascertainment of MetS

The presence of the MetS was determined at baseline using the National Cholesterol Education Program Third Adult Treatment Panel guidelines (National Cholesterol Education Program (NCEP)., 2002). A person is identified as having MetS if they meet 3 or more of the following criteria: (1) abdominal obesity (waist girth ≥ 102 cm for male or ≥88 cm for female); (2) hypertriglyceridemia (triglycerides level ≥150 mg/dl; (3) low HDL-C level (HDL <40 mg/dl for men or <50 mg/dl for women; (4) hypertension (systolic blood pressure ≥130 mm Hg and diastolic blood pressure ≥85 mm Hg or currently taking an antihypertensive medication); and (5) high fasting glucose level (fasting glucose level ≥110 mg/dl or taking antidiabetic medication).

### Cognitive Function Assessment

CARDIA technicians who underwent formal training and certification administered a battery of cognitive tests at the 5-year follow-up visit that included processing speed (Digit Symbol Substitution Test, DSST), executive function (the Stroop Test), verbal memory (Rey Auditory Verbal Learning Test, RAVLT), verbal fluency (category and letter fluency), and global cognitive function (Montreal Cognitive Assessment, MoCA). Moreover, we created a “global cognition” score by combining the z-scores (mean 0, SD 1) of the DSST, RAVLT, Stroop, and verbal fluency tests.

### White Matter Integrity

Structural MRI and diffusion tensor imaging (DTI) scans were completed in 3 Tesla (3 T) magnetic resonance scanners (Philips 3 T Achieva/2.6.3.6 platform in Birmingham, AL; Siemens 3 T Tim Trio/VB 15 platform in Minneapolis, MN; and Siemens 3 T Tim Trio/VB 15 platform in Oakland, CA) using standardized protocols at the 5-year follow-up visit (2015–2016) on a subsample of participants (*n* = 453). More detailed neuroimaging methods were previously reported (Launer et al., 2015). The DTI was used to compute voxel-wise maps of white matter integrity, as indexed by fractional anisotropy (FA) and mean diffusivity (MD). The FA measure estimates the degree or uniformity to which water diffuses along the direction of myelinated tracks in the white matter. The FA scores range from 0 to 1 with lower scores indicating worse white matter integrity. The MD measure reflects the diffusivity of water such that the reduced tissue density is associated with a higher MD, indicating worse white matter integrity. We examined the white matter integrity (FA, MD) overall and by lobe, such that the mean values overall and within specific lobes were averaged across the left and right hemispheres.

### Covariates

The demographic characteristics, current cigarette smoking status, and alcohol use in ml/week were based on self-report at baseline. Self-reported annual family income was measured on a 9-point scale, with 1 = < $5,000, 5 = $25,000–$34,999, and 9 = $100,000 or more, and dichotomized as income above or below the median income category $35,000 through $49,999. The participants were classified according to *APOE* phenotype and categorized as having any epsilon 4 (ε4) vs. no ε4 allele. The Center for Epidemiological Studies Depression scale (CESD) was used to assess depressive symptoms (Radloff, 1977). The C-reactive protein (CRP) levels were measured from blood sample at baseline, dichotomized as high (above 3.0 mg/L) vs. normal (below 3.0 mg/L). Covariates were chosen on the basis of prior evidence of variables that can either be considered the cause of the exposure, the outcome, or both. Inflammation has been suggested to be a moderating factor between MetS and neurocognitive outcomes (Petersen et al., 2006; Romero et al., 2009) and is associated with lower white matter integrity (Whitmer et al., 2005). Smoking is known to contribute to cardiovascular disease and has been identified as one of the midlife vascular risk factors that accelerate structural brain aging and cognitive decline (Yaffe et al., 2007).

### Statistical Analysis

The characteristics of the study population by MetS at baseline were compared using the χ^2^-tests for categorical variables and the one-way ANOVA for continuous variables.

Using linear regression, we examined the association between MetS and performance in cognitive domains at the 5-year follow-up visit. All models were first examined unadjusted for covariates, then adjusted for age, sex, education, race, income, and further adjusted for smoking, alcohol consumption, and depressive symptoms. Moreover, we tested for interactions between MetS and sex, race, *APOE* ε4, and CRP high/normal, respectively.

We further examined the association between MetS and white matter integrity measures FA and MD in the CARDIA MRI sample using a linear regression. These models were adjusted for age, sex, education, race, and scanning center.

Lastly, we examined the associations between white matter integrity measures and the cognitive tests using a linear regression.

All analyses were completed with STATA version 15.1, and R 4.0.4. The significance testing was two sided with the significance level set at *p* < 0.05. As sensitivity analyses, we further adjusted the *p*-values for multiple testing in the MetS and cognitive models and the MetS and white mater integrity models using Bonferroni correction.

## Results

Among the 2,880 participants, the mean age at our study baseline was 50 (SD = 3.6), 1,334 (46.3%) were Black, and 1,644 (57.1%) were women. Participants with MetS (*n* = 534, 18.5%) were more likely to be women, be of Black race, have lower education, lower income, consume more alcohol, have more depressive symptoms, to be an *APOE* ε4 carrier, and to have high CRP (Table 1).

**Table 1:** Characteristics of 2,880 CARDIA participants by metabolic syndrome at midlife.

**Variable mean (sd), *n* (%)**	**No MetS** **(*n* = 2,346, 81.5%)**	**MetS** **(*n* = 534, 18.5%)**	***p*-value**
Age, y	50.1 (3.6)	50.5 (3.6)	0.055
Female	1,361 (58.0)	284 (53.2)	0.042
Education, y	14.2 (2.3)	13.6 (2.1)	<0.001
Black	1,053 (44.9)	281 (52.6)	0.001
Alcohol: ml/week	86.4 (171.8)	59.2 (117.0)	<0.001
Current smoking	349 (15.1)	94 (17.9)	0.107
Income below median^a^	739 (31.5)	226 (42.3)	<0.001
*APOE* ε4 carrier	592 (28.6)	170 (36.3)	0.001
Depression: CESD > 16	475 (20.4)	132 (25.0)	0.020
High CRP	405 (18.9)	203 (44.0)	<0.001
**MetS components**			
Waist girth (cm)	90.5 (14.1)	109.2 (13.6)	<0.001
triglycerides (mg/dL)	94.9 (49.8)	189.6 (144.3)	<0.001
glucose level (mg/dL)	93.7 (17.8)	119.5 (43.9)	<0.001
Systolic BP (mm Hg)	116.3 (14.3)	126.0 (16.4)	<0.001
Diastolic BP (mm Hg)	72.2 (10.4)	79.8 (10.8)	<0.001
HDL (mg/dl)	61.3 (17.2)	44.5 (11.9)	<0.001

*CARDIA, Coronary Artery Risk Development in Young Adults; MetS, Metabolic Syndrome; APOE, Apolipoprotein epsilon 4; CESD, Center for Epidemiological Studies Depression scale; High CRP, C-reactive protein level over 3.0 mg/L. BP, blood pressure*.

a *Self-reported annual family income was measured on a 9-point scale, with 1 = < $5,000, 5 = $25,000–$34,999, and 9 = $100,000 or more. Median range of income: $35,000 through $49,999*.

Those with MetS had worse performance at the 5-year follow-up visit on the RAVLT (β: −0.37, 95% CI: −0.66 to −0.09), DSST (β: −2.22, 95% CI: −3.54 to −0.88), verbal fluency (β: −0.77, 95% CI: −1.48 to −0.05), and the stroop tests (β: 1.25, 95% CI: 0.20 to 2.30) but not on the MoCA, after adjusting for age, sex, education, income, and race. Moreover, MetS was associated with worse performance on the global cognitive composite (Table 2). After adjusting the *p*-value for multiple testing (*p* = 0.01), only the DSST and RAVLT remained significantly associated with MetS status. With further adjustment for depressive symptoms, smoking, and alcohol consumption, MetS remained significantly associated with lower performance on the RAVLT, stroop and DSST, but not on verbal fluency.

**Table 2:** The association between midlife metabolic syndrome and cognitive performance among the 2,880 CARDIA participants.

		**β-coefficient (95% CI)**	
	**Mean (SD)**	**Unadjusted model**	**Adjusted model^**a**^**
**RAVLT delay**
No MetS	8.7 (3.4)	Reference	Reference
MetS	7.8 (3.4)	−0.88 (−1.20 to −0.56)	−0.37 (−0.66 to −0.09)^*^
**DSST**
No MetS	69.1 (16.6)	Reference	Reference
MetS	63.7 (16.6)	−5.41 (−6.98 to −3.84)	−2.22 (−3.54 to −0.88)^*^
**Stroop Test**
No MetS	22.2 (11.1)	Reference	Reference
MetS	24.8 (14.1)	2.62 (1.50 to 3.74)	1.25 (0.20 to 2.30)
**Verbal Fluency**
No MetS	31.5 (8.3)	Reference	Reference
MetS	29.6 (7.9)	−1.91 (−2.70 to −1.13)	−0.77 (−1.48 to −0.05)
**MoCA**
No MetS	24.1 (3.9)	Reference	Reference
MetS	23.1 (3.9)	−0.93 (−1.29 to −0.56)	−0.21 (−0.52 to 0.09)
**Global composite score (z-score)**
No MetS	0.06 (0.7)	Reference	Reference
MetS	−0.18 (0.7)	−0.25 (−0.31 to −0.18)	−0.10 (−0.15 to −0.05)

*CARDIA, Coronary Artery Risk Development in Young Adults; CI, confidence interval; RAVLT, Rey Auditory Verbal Learning Test; DSST, Digit Symbol Substitution Test; MoCA, Montreal Cognitive Assessment*.

a *Adjusted for age, sex, education, race, and income*.

*Lower scores indicate worse performance in all tests except the Stroop test, where higher scores indicate worse performance*.

* *Significant after adjusting for multiple testing (p: 0.01)*.

We did not find evidence for an interaction between MetS and sex, race, CRP, or *APOE* ε4 status, respectively.

**Figure 1:**
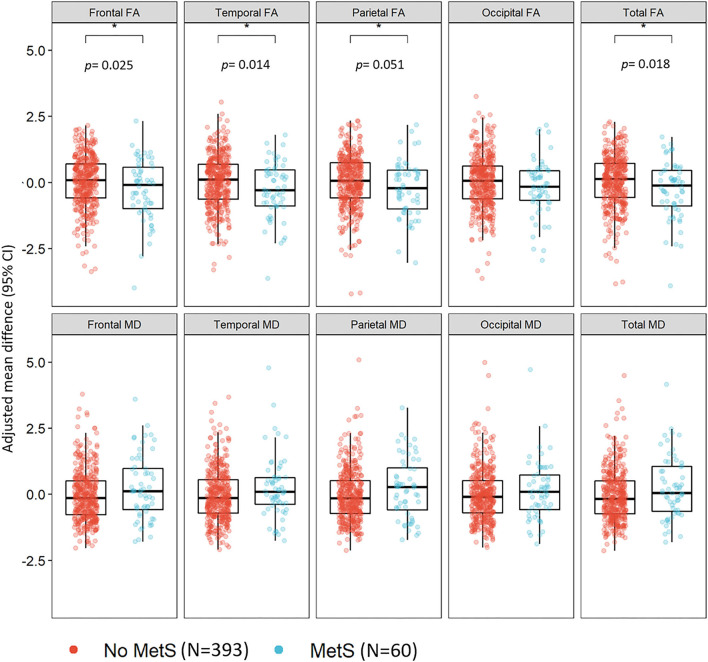
The association between the metabolic syndrome and white matter integrity in midlife among 453 CARDIA participants. CARDIA, Coronary Artery Risk Development in Young Adults; CI, confidence interval; FA, fractional anisotropy; MD, mean diffusivity; MetS, metabolic syndrome. Models adjusted for age, sex, education, race, and scanning center.

In the MRI sub-sample, MetS (*n* = 60, 13.2%) was associated with lower frontal (β: −0.30, 95% CI: −0.55 to −0.04), temporal (β: −0.32, 95% CI: −0.57 to −0.07), parietal (β: −0.28, 95% CI: −0.55 to −0.04), and total FA (β: −0.32, 95% CI: −0.58 to −0.06), adjusted for age, sex, education, and scanning center. The associations did not survive adjusting the *p*-value for multiple testing (*p* = 0.01); the *p*-values are shown in Figure 1. There was a tendency for MD values to be higher in the MetS group; however, the differences were not significant (Figure 1).

Lastly, we tested the association between regional and total FA and the cognitive tests. Higher values of frontal, parietal, and total FA were significantly associated with better performance on all cognitive tests; temporal FA was significantly associated with better performance on the DSST, verbal fluency, and MoCA; and occipital FA was significantly associated with a better performance on the DSST and verbal fluency (*p* < 0.05; Supplementary Table 1).

## Discussion

In this biracial cohort of middle-aged community-dwelling adults, we found that having MetS in midlife is associated with worse cognition in most domains and with reduced WM integrity. These results add to the evidence that MetS may be harmful to cognitive function even as early as midlife. Moreover, our findings suggest that MetS may contribute to early reduced WM integrity and microstructural damage.

Our results align with literature on the contribution of CVRFs to the risk of late-life cognitive decline and dementia (Whitmer et al., 2005; Yaffe et al., 2007, 2009; Romero et al., 2009) and with a small but growing literature demonstrating the importance of constellations of CVRFs such as MetS, for cognitive performance in earlier stages of the life course (Kazlauskaite et al., 2020; Yaffe et al., 2020; Foret et al., 2021). The findings of this study are consistent with several studies in older adults showing that MetS increases the risk for cognitive impairment in late life (Yaffe et al., 2007, 2009). In contrast, our investigation evaluated the midlife exposure to MetS and cognitive performance in several cognitive domains 5 years later. These results indicate an early divergence of cognitive performance in middle-aged adults with MetS. As midlife is a sensitive time period where cognitive changes start to occur, lower cognitive performance during this time period may influence cognitive trajectories into late life. The clinical significance is that midlife may be the most optimal window for intervention in people with MetS, before more significant damage occurs.

Growing evidence suggests that there is an association between MetS and impairment of WM integrity and microstructural damage. Case-control studies have reported reduced FA in patients with MetS compared to controls (Shimoji et al., 2013; Alfaro et al., 2016). One prospective study with participants ranging from midlife to old age found that higher vascular burden was associated with lower FA and higher MD in many WM structures (Williams et al., 2019). However, most of these studies have been case-control studies or observational studies with wide age ranges. In this study, we found that MetS is associated with widely reduced WM integrity in midlife. These findings suggest that MetS, even at a midlife, could be related to cognitive changes, thereby increasing the risk of dementia. Indeed, lower FA values were associated with lower cognitive scores in this study which supports this hypothesis, However, MetS is a cluster of conditions that represent a high-yield target for interventions, and as midlife is a sensitive period where age-related cognitive changes begin, the optimum time for intervening may be before or during midlife.

The clustering of CVRFs such as in MetS may result in structural and functional changes in vasculature, culminating in cerebral small vessel disease, potentially affecting cerebral blood flow (Romero et al., 2009). Such changes may present themselves early as reduced WM integrity.

The strengths of this study include the well-characterized longitudinal sample of Black and White adults, in which we were able to evaluate the relationship of midlife MetS exposure with cognitive performance and WM integrity 5 years later. However, there are also limitations to consider. The CARDIA cohort is a select sample by year 25 which was our study's baseline, and therefore subject to selective attrition. In addition, the MRI sample was healthier than the main CARDIA sample and had lower MetS prevalence by 5%. Such sample selection may have underestimated the association. Moreover, the MRI and cognitive measures in this study were from a single point in time, as such there is no baseline assessment of cognitive status or MRI. Therefore, the available data do not allow for making inferences about causal relationships, and our findings need to be confirmed with additional follow-up data.

This study links midlife MetS to lower cognitive performance and WM integrity in midlife. Our study provides evidence for the need to target adults at risk earlier in the lifespan; middle-aged adults with MetS may represent a group at risk that may benefit from early monitoring and education.

## Data Availability Statement

Publicly available datasets were analyzed in this study. This data can be found here: https://www.cardia.dopm.uab.edu/.

## Ethics Statement

At each examination, participants provided written informed consent, and study protocols were approved by Institutional Review Boards at each study site (Kaiser Permanente Division of Research, Oakland Field Center; Northwestern University, Feinberg School of Medicine, Department of Preventive Medicine, Chicago Field Center; University of Minnesota, School of Public Health, Division of Epidemiology and Community Health, Minneapolis Field Center; University of Alabama at Birmingham, School of Public Health, Department of Epidemiology, Birmingham Field Center) and the CARDIA Coordinating Center.

## Author Contributions

CD is responsible for study conceptualization, data analysis, and drafting of the manuscript. TH is responsible for intellectual input and revision of the manuscript. NA is responsible for providing clinical expertise and revision of the manuscript. SS and KY is responsible for data acquisition and revision of the manuscript. All authors contributed to the article and approved the submitted version.

## Funding

The Coronary Artery Risk Development in Young Adults Study (CARDIA) was supported by Contracts HHSN268201800003I, HHSN268201800004I, HHSN268201800005I, HHSN268201800006I, and HHSN268201800007I from the National Heart, Lung, and Blood Institute (NHLBI). CARDIA was also partially supported by the Intramural Research Program of the National Institute on Aging (NIA) and an intra-agency agreement between NIA and NHLBI (AG0005). The CARDIA Cognitive Ancillary Study was supported by the National Institute on Aging Grant R01 AG063887-01 (NIA Multiple-PI: KY and SS). This research was also supported by the NIA Grant K24 AG031155 (PI: KY) and an Alzheimer's Association Grant AARF-21-851960 (CD).

## Conflict of Interest

SS was employed by Kaiser Permanente Northern California. The remaining authors declare that the research was conducted in the absence of any commercial or financial relationships that could be construed as a potential conflict of interest.

## Publisher's Note

All claims expressed in this article are solely those of the authors and do not necessarily represent those of their affiliated organizations, or those of the publisher, the editors and the reviewers. Any product that may be evaluated in this article, or claim that may be made by its manufacturer, is not guaranteed or endorsed by the publisher.
