# Supplementary material for: The Metabolic Syndrome Is Associated With Lower Cognitive Performance and Reduced White Matter Integrity in Midlife: The CARDIA Study
Source: Front Neurosci. 2022 Jul 18;16:942743. doi: 10.3389/fnins.2022.942743 (PMC9339689; doi:10.3389/fnins.2022.942743)
Supplement: Supplementary file 1 [file Table_1.DOCX]

**Supplementary Table 1.** The association between midlife fractional anisotropy in midlife and cognitive performance among the 453 CARDIA participants.

| Cognitive test |  |  |  |  |  |
| --- | --- | --- | --- | --- | --- |
|  | **β- coefficient (95% CI)** | | | | |
|  | Frontal FA | Temporal FA | Parietal FA | Occipital FA | Total FA |
| RAVLT delay | 0.40 (0.09 to 0.71)^**^ | 0.31 (-0.01 to 0.63) | 0.35 (0.06 to 0.65)^*^ | 0.15 (-0.15 to 0.45) | 0.35 (0.04 to 0.66)^*^ |
| DSST | 3.02 (1.69 to 4.36)^**^ | 2.92 (1.53 to 4.32)^**^ | 2.50 (1.21 to 3.79)^**^ | 2.16 (0.86 to 3.45)^**^ | 3.08 (1.75 to 4.42)^**^ |
| Stroop Test | -2.22 (-3.26 to -1.19)^**^ | -1.08 (-2.17 to 0.02) | -1.32 (-2.32 to -0.31)^**^ | -0.88 (-1.89 to 0.13) | -1.77 (-2.81 to -0.72)^**^ |
| Verbal Fluency | 1.31 (0.59 to 2.03)^**^ | 1.17 (0.42 to 1.93)^**^ | 1.30 (0.62 to 1.99)^**^ | 0.76 (0.07 to 1.45)^*^ | 1.43 (0.63 to 2.07)^**^ |
| MoCA | 0.59 (0.28 to 0.89)^**^ | 0.41 (0.10 to 0.73)^**^ | 0.43 (0.15 to 0.72)^**^ | 0.27 (-0.03 to 0.56) | 0.55 (0.25 to 0.85)^**^ |

CARDIA, Coronary Artery Risk Development in Young Adults; CI, confidence interval; RAVLT, Rey Auditory Verbal Learning Test; DSST, Digit Symbol Substitution Test; MoCA, Montreal Cognitive Assessment.

^a^Adjusted for age, sex, education, race, and income.

Lower scores indicate worse performance in all tests except the Stroop test, where higher scores indicate worse performance.

^*^p<0.05

^**^p<0.01
